# The relationship between burden and caregiver’s sleep disturbances in dementia: a systematic review

**DOI:** 10.1590/1980-5764-DN-2023-0030

**Published:** 2023-12-15

**Authors:** Bárbara Cristina da Costa Monteiro, Tatiana Teresa Belfort Almeida dos Santos, Marcela Moreira Lima Nogueira, Marcia Cristina Nascimento Dourado

**Affiliations:** 1 Universidade Federal do Rio de Janeiro, Instituto de Psiquiatria, Centro de Doença de Alzheimer, Rio de Janeiro RJ, Brazil.

**Keywords:** Sleep Quality, Caregivers, Caregiver Burden, Dementia, Sleep Wake Disorders, Sleep, Qualidade do Sono, Cuidadores, Fardo do Cuidador, Demência, Transtornos do Sono-Vigília, Sono

## Abstract

Caregivers of people living with dementia (PLwD) have a high burden degree that leads to health issues, including sleep.

**Objective:**

This study aimed to analyze the impacts of the caregiving burden on caregiver’s sleep disturbances.

**Methods:**

This systematic review involved a qualitative analysis of publications on Web of Science and Pubmed/Medline databases published between February 2018 and August 2022.

**Results:**

A total of 27 studies were identified and analyzed. Caregiver’s sleep presents impairments in sleep latency, sleep fragmentation, sleep duration, subjective sleep quality, daytime dysfunction, and insomnia. Caregiver’s distress and depressive symptoms have a dual relationship with sleep problems.

**Conclusion:**

Sleep disturbances presented by caregivers are correlated with higher burden levels and lead to more vulnerability to psychiatric symptoms and health issues.

## RESUMO

Cuidadores de pessoas com demência possuem alto grau de sobrecarga que impacta de forma objetiva o sono e suas dimensões.

**Objetivo:**

Este estudo teve o objetivo de analisar o impacto da sobrecarga nas alterações do sono do cuidador.

**Métodos:**

Esta revisão sistemática envolveu a análise de resultados quantitativos e qualitativos de publicações das bases de dados Web of Science e PubMed/ Medical Literature Analysis and Retrieval System Online (Medline) publicadas entre fevereiro de 2018 e agosto de 2022.

**Resultados:**

O total de 27 estudos foi identificado e analisado. Cuidadores apresentam prejuízos na latência, fragmentação, duração e qualidade subjetiva do sono, disfunção diurna e insônia. O estresse e sintomas depressivos apresentados pelo cuidador possuem com o sono uma relação bidirecional.

**Conclusão:**

Os distúrbios do sono apresentados pelos cuidadores estão correlacionados com o alto nível de sobrecarga e geram maior vulnerabilidade para sintomas psiquiátricos e problemas de saúde.

## INTRODUCTION

Global life expectancy increases every year and, with this, the number of people with dementia. Caregivers of people living with dementia (PLwD) are often relatives or close friends who spend one decade in the care^1^ and, despite the disinformation about the symptoms, are mainly responsible for its management^2^, generally experiencing burden. The term “burden” refers to the impact that providing care for a family member has on a caregiver’s life^3^. This population can experience impacts on mental, physical, emotional, and financial health^4^. Generally, burden has a mutual impact on sleep and high stress levels, leading to depression, anxiety, and poor sleep quality^5,6^.

Sleeping is crucial to maintain emotional and physical health, but nearly 63% of family caregivers are affected by chronic insomnia^7^, which predicts caregiver strain^1^. The sleep average of caregivers is 6.5 hours per night^8,9^, contrary to the recommendation of the National Sleep Foundation for older adults that determines 8 hours of sleep for optimal wellbeing^10^. The total hours of sleep are impacted by the nighttime care needed, leading to exhaustion the next day and daytime impairment^2,11–13^.

Sleep disorders affect 50 to 70% of caregivers of PLwD^14^, resulting in long sleep onset latency, wake-after sleep onset, short sleep duration, low sleep efficiency^15,16^, changes in central stress, low sleep quality^17^, daytime sleepiness, poor self-rated sleep^18^, and sleep fragmentation^19^. These disturbances can negatively impact the immune system, elevate stress hormones, and increase the risk for cardiovascular diseases^20^, and the correlation between sleep and depressive symptoms predicts increased body mass index^21^, elevated coagulation, and inflammatory levels^22^.

This systematic review aimed to provide updated evidence on the relationship between caregivers’ burden and sleep patterns. Specifically, the review focused on how caregiving impacts sleep quality and perceptions of sleep over the past five years.

## METHODS

This systematic review was conducted following the methodology suggested by the Preferred Reporting Items for Systematic Reviews and Meta-Analyses (PRISMA)^23^. Literature research was carried out during January of 2023 using Web of Science and Pubmed/Medline databases. Search keywords were “Caregivers sleep AND Dementia,” “Caregiver AND Sleep Problems,” “Insomnia AND Caregivers,” “Sleep Quality AND Dementia,” “Sleep Quality AND Caregivers,” “Sleep AND Caregivers,” and “Dementia AND sleep.”

Inclusion criteria were:

- cross-sectional and longitudinal, randomized and non-randomized studies;
- studies with caregivers of PLwD;
- studies that included sleep in the outcomes.

Exclusion criteria were:

- studies with etiologies other than dementia;
- case reports;
- meta-analyses and systematic reviews; and
- studies with inpatients.

The authors read the selected abstracts and, when there was not enough information in the abstract to determine inclusion and exclusion criteria, the full text was retrieved. Two authors then independently reviewed the complete publications of the remaining papers and reached a consensus regarding inclusion criteria. The included studies were categorized according to their design, sample, method, and results. All selected articles were published between February 2018 and August 2022 and were in English. Data extraction occurred between February 2023 and April 2023.

To help analyze the quality of the articles found, the Mixed Methods Appraisal Tool (MMAT)^24^ was used. MMAT is a tool that helps to identify the methodological quality of qualitative research, randomized controlled trials, non-randomized studies, quantitative descriptive studies, and mixed method studies. In the results table, every item evaluated in the analysis has a rate between 1 and 7.

**Figure 1:**
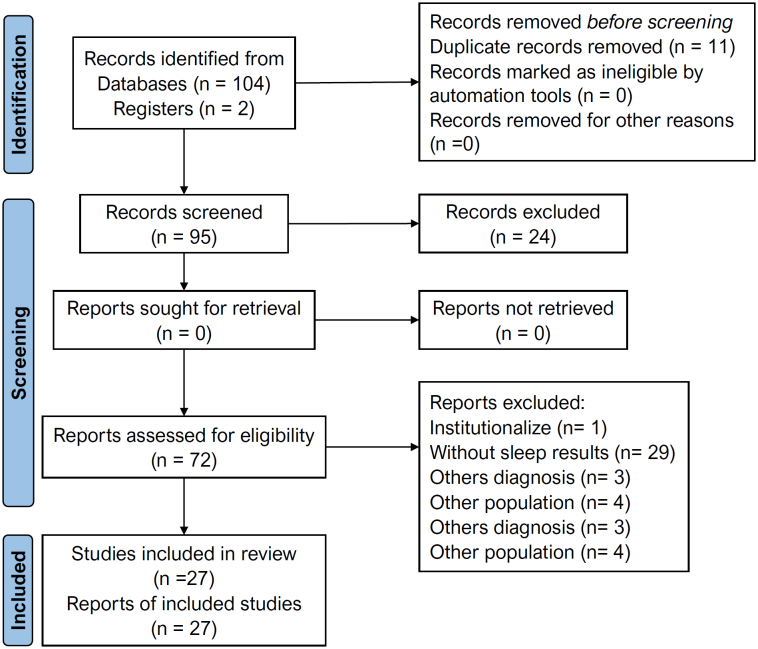
Flowchart of search and review process.

Initial screening was performed by conferring the publication date and reading the title and abstracts. Articles that did not meet inclusion criteria were excluded, and those that were possibly eligible were retained, then read in entirety to confirm eligibility. The reasons for the article’s exclusion were registered in the PRISMA flowchart (Figure 1).

Information collected through sleep and burden scales and objective measures of altered sleep patterns obtained through polysomnography and actigraphy were considered. Caregivers’ characteristics were obtained from sixteen articles that specified gender, relationship with the care recipient, time spent on tasks, and age.

This systematic review was registered in the International Prospective Register of Systematic Reviews (PROSPERO) under CRD42023392955.

## RESULTS

One hundred and four articles were found in the databases, and two registers were identified through Google Scholar. After removing duplicates, ninety-five articles were selected for the analysis according to inclusion criteria. Inclusion/exclusion criteria excluded seventy-two articles, remaining twenty-seven articles for final inclusion, with 19,859 caregivers analyzed. Table 1 depicts the study’s main characteristics and results.

**Table 1:** Studies’ main characteristics and results.

Study	Design	Sample/Mean age	Objective	Sleep measures instruments	Others instruments	Results	MMAT
Bao et al.^25^ (2022)	Descriptive Longitudinal	177 individuals(61.3 years)	To investigate the effects of the pandemic on anxiety, depression and care burden in caregivers of nursing patients with AD, DLB, and MCI, over a one-year period.	PSQI	ZBI GAD-7 PHQ-9	Sleep disturbances after lockdown in Covid-19 pandemic worsened caregiver burden and psychological status.	5
Blinka et al.^26^ (2022)	Descriptive Cross-sectional	498 individuals (69.2 years)	To evaluate differences in self-reported sleep quality between caregivers and a control group	Self-report	CES-D	Caregivers show long sleep onset latency.	5
Brewster et al.^7^ (2022)	Descriptive cross-sectional	28 individuals (65.1 years)	To evaluate sleep quality in caregivers of PLwD and correlate it with cognitive function.	Sleep Diary	–	Caregivers of PLwD showed high amounts of wake after sleep onset and lower sleep efficiency	5
Bussè et al.^27^ (2022)	Descriptive Longitudinal	151 individuals (62 years)	To assess the severity of long-term stress-related symptoms in caregivers of PLwD one year after the COVID-19 pandemic	PSQI	DASS-21 COPE-NVI	96% of caregivers presented sleep alterations between the four frequently stress-related symptoms	5
Kim and Cha ^4^ (2022)	Descriptive Cross-sectional	399 individuals (<65 - >85 years)	To identify the mediating effects of sleep quality on the relationship between perceived stress and HRQoL among primary caregiving spouses of patients with severe dementia	PSQI	PSS EQ-5DPHQ-9	Caregiving spouses of patients with severe dementiapresented low sleep quality	6
Liu et al. ^13^ (2022)	Descriptive Cross-sectional	1,073 individuals (61.9 years)	To examine sleep, negative effects, and stress biomarkers from the perspective of stressor exposure and reactivity.	Self-reported Sleep Diary	Non-Specific Psychological Distress Scale Daily Stress and Health	When caregivers have more time in bed, they present lower cortisol levels and anxiety symptoms	5
Lu et al.^2^ (2022)	Descriptive Cross-sectional	269 individuals (≥60 years)	To evaluate caregiver distress between older adults with dementia living in the community and in nursing homes	Sleep domain of NPI	NPI	Sleep disorders are responsible for the most frequent “very severe” caregiver distress	6
Osakwe et al.^19^ (2022)	Descriptive Cross-sectional	1,142 individuals (58.5 years)	To examine predictors of sleep disturbance and strain among caregivers of PLwD.	Self-report	Reported measures	Caregivers with greater sleep difficulty were more likely to report high blood pressure.	6
Pinyopornpanish et al.^16^ (2022)	Descriptive cross-sectional	102 individuals (55 years)	To explore the association between BPSD and caregiver stress, burden, and depression.	Sleep domain of NPI	PHQ-9 ZBI-22 PSS NPI	Caregivers presented sleep disorders and night-time behaviors that impacted caregiver burden and lead to depressive symptoms.	6
Sanprakhon et al.^6^ (2022)	Descriptive Cross-sectional	72 individuals (>18 years)	To examine the relationships between caregiving stress and sleep quality among family caregivers of older adults with dementia.	PSQI	RSS	Caregiving stress is positively correlated with poor sleep quality	5
Vara-Garcia et al.^5^ (2022)	Descriptive Longitudinal	111 individuals (74.5 years)	To analyze the possible longitudinal effects of various cognitive and behavioral variables on caregivers’ depressive symptoms.	PSQI	WCQ CSES PMS PES-AD PANAS CESD-10	Caregivers with impacts on sleep quality presented higher levels of depressive symptoms	6
Xu et al.^15^ (2022)	Clinical Trial randomized	71 individuals (54 years)	To test an evidence-based behavioral activation protocol to improve sleep quality in caregivers of PLwD.	PSQI	CES-D	Caregivers had improvement in sleep quality, more perception of positive aspects of caregiving and reduction of depressive symptoms.	5
Hoyt et al.^18^ (2021)	Descriptive Cross-sectional	35 individuals (21.1 years)	To examine subjective and objective indicators of sleep quality and diurnal cortisol rhythms among young adult caregivers relative to non-caregiving peers.	PSQI	LCI HADS	Caregivers exhibit more sleep disturbance, greater sleep latency, and more sleep fragmentation.	5
Jiménez-Gonzalo et al.^17^ (2021)	Descriptive Cross-sectional	271 individuals (62.9 years)	To analyze the psychometric properties of the ISI in a Spanish dementia caregiver sample.	ISI	POMS CES-D	ISI was significantly associated with lower sleep quality, less average sleep time per day, and lower self-perception of vitality, as well as with higher depressive and anxiety symptoms.	5
Jiménez-Gonzalo et al.^22^ (2021)	Descriptive Cross-sectional	264 individuals (62.7 years)	To explore the association between caregiver sleep problems and behavioral problems in care recipients.	ISI	–	Disrupted behaviors in care recipients are associated with poorer sleep of caregivers.	5
Martínez-Santos et al.^28^ (2021)	Multi-centric cross-sectional prospective	423 individuals (59.9 years)	Describe the care provided by family caregivers of people with dementia and the impact on their health	Non-Specified	ICUB97-R questionnaire	Most complaints by caregivers are about sleep time or resting less.	7
Rigby et al.^29^ (2021)	Descriptive cross-sectional	515 individuals (61.4 years)	To examine the differences in the caregiver experience between DLB, PDD, and AD	Non-Specified	RMBPC CGI ZBI QoL PCI PWB PHQ-2	Caregiver experience of burden depends on the sleep characteristics presented.	6
Sato et al.^30^ (2021)	Descriptive cross-sectional	126 individuals (64.1 years)	To compare self-efficacy in DLB Caregivers and AD Caregivers	ISI	ZBI BDI-II RSCSE	DLB caregivers present more sleep problems than AD caregivers	6
Song and Kim ^31^ (2021)	Descriptive Cross-sectional	11,591,278 individuals (55.3 years)	To compare sleep quality between cohabitating caregivers, noncohabitating caregivers, and noncaregivers of PLwD.	PSQI	–	Cohabitating caregivers showed poorer sleep quality when compared to noncohabitating caregivers. Noncohabitating caregivers have poorer sleep quality when compared to noncaregivers.	6
Corey et al.^32^ (2020)	Descriptive Cross-sectional	171 individuals (>18 years)	To explore the independent associations of sociodemographic variables, personality and coping, environmental variables, and caregiver guilt with the sleep quality of former family caregivers of PLwD following care recipient’s death.	PSQI	BFI BRIEF-COPE CES-D DASS	Personality traits, pre-loss depression, and copping strategies are directly associated with sleep quality.	6
Liang, Aranda and Lloyd ^9^ (2020)	Descriptive Cross-sectional	669 individuals (60.3 years)	To explore the association between sleep maintenance insomnia and role overload.	SMI	SPM	Caregivers presents high rates of sleep disturbance.	6
Ryuno et al.^12^ (2020)	Descriptive Longitudinal	23 objects (66.9 years)	To identify the association between care burden and objective/subjective sleep quality	PSQI	ZBI	Care burden is significantly associated with total sleep time and PSQI score.	7
Simón et al.^33^ (2019)	Descriptive Cross-sectional	293 individuals (56.2 years)	To examine the relationship between caregiver burden and sleep quality.	PSQI	CBI	Caregiver burden is significantly associated with sleep quality.	6
Eleuteri et al.^21^ (2018)	Descriptive Cross-sectional	117 individuals (54.3 years)	To examine the association between sleep quality and BMI in a population of caregivers of PLwD.	PSQI	Eating Behavior Questionnaire	Caregivers with low depressive symptoms and poor sleep quality have higher BMI scores. Women presents higher BMI scores when compared with males.	6
Legget et al.^11^ (2018)	Descriptive Cross-sectional	451 individuals (59.1 years)	To examine caregiver health and psychological wellbeing as predictors of nighttime awakenings.	5-point scale item	–	16% of caregivers reported nighttime awakening every night. Emotional caregiving difficulties predict nighttime awakenings.	4
Liu et al.^34^ (2018)	Descriptive Cross-sectional	492 individuals (58.2 years)	To compare caregiver burden and sleep quality of patients with frontotemporal lobar degeneration, DLB and AD.	PSQI	ZBI PHQ-9 GAD-7	Caregivers of frontotemporal lobar degeneration and DLB experience more burden and sleep impairments.	6
Polenick et al.^14^ (2018)	Retrospective Analysis Cross-sectional	104 individuals (75.5 years)	To evaluate the association between caregivers’ medical/nursing tasks.	Self-report	–	Caregivers who performed a higher number of medical/nursing tasks reported significantly more frequent care-related sleep disturbances	4

Abbreviations: AD, Alzheimer Disease; DLB, Dementia with Lewy Bodies; MCI, Mild Cognitive Impairment; PSQI, Pittsburgh Sleep Quality Index; ZBI, Zarit Burden Interview Short Version; GAD-7, Generalized Anxiety Disorder Scale; PHQ-9, Patient Health Questionnaire-9; CES-D, Center for Epidemiological Scale-Depression; PLwD, People Living with Dementia; DASS-21, Depression, Anxiety and Stress Scale; COPE-NVI, Coping Orientation to the Problems Experienced; HRQoL, Health-related Quality of Life; PSS, Perceived Stress Scale; EQ-5D, European Quality of Life Five Dimension; NPI, Neuropsychiatric Inventory; BPSD, Behavioral and Psychological Symptoms of Dementia; RSS, Relative Stress Scale; WCQ, Ways of Coping Questionnaire; CSES, Coping Self-Efficacy Scale; PMS, Personal Mastery Scale; PES-AD, Pleasant Events Schedule−AD; PANAS, Positive and Negative Affect Schedule; CESD-10, Center for Epidemiological Scale-Depression, 10 Item Version; LCI, Level of Care Index; HADS, Hospital Anxiety and Depression Scale; ISI, Insomnia Severity Index; POMS, Profile of Mood States; ICUB97-R, Data-gathering instrument based on Virginia Henderson’s 14 Needs nursing model; PDD, Parkinson Disease Dementia; RMBPC, Revised Memory and Behavioral Problems Checklist; CGI, Marwit-Meuser Caregiver Grief Inventory; QoL, Quality of Life in Alzheimer Disease; PCI, The Perceived Change Index; PWB, Ryff Psychological Well-Being Scale; PHQ-2, Health Questionnaire 2-item Depression Scale; BDI-II, Beck Depression Inventory II; RSCSE, Revised Scale for Caregiving Self-Efficacy; BFI, Big Five Inventory; BRIEF-COPE, Self Report Questionnaire to Measure Cope Strategies in Stressful Life Events; SPM, Stress Process Model of Caregiving; CBI, Caregiver Burden Inventory SMI, SleepMed Insomnia Index.

### Studies design

Among the studies analyzed, four were descriptive longitudinal^4,12,25,27^, twenty were descriptive cross-sectional^2,4–7,9,11,13,16–19,21,26,29–34^, one was a randomized clinical trial^15^, one was a multi-centric cross-sectional prospective^28^, and one was a retrospective cross-sectional analysis^14^.

### Participants

### Caregivers’ characteristics

Studies have shown that most caregivers were women and family members, mainly spouses and adult children^6,8,12,14–17,22,29,31,33,34^, usually living with the care recipient^12,17,18,22,25,31^ (Table 2).

**Table 2:** Caregivers characteristics.

Characteristic		Result (%)
Gender	Female	69.5
Relationship	Partner	18.5
	Children	33.3
Living with care recipient		71.3
Time spent in care	In years	5.7
	In daily hours	14.3
Age range (y)		60.3

Caregivers usually spend 14.3 hours per day in care tasks^13,18,19,22,33^, being responsible for the PLwD for nearly 5.7 years^1,14,17,22,25,31,32,33^. The mean age of caregivers in the selected studies was 60.3 years.

### Instruments

The most common instrument used to assess sleep quality was the Pittsburgh Sleep Quality Index (PSQI)^1,3,5,11,14,18,21,25,27,28,32–34^. This golden self-rated questionnaire analyzes seven dimensions of sleep quality: subjective sleep quality, sleep latency, sleep duration, habitual sleep efficiency, sleep disturbances, use of sleeping medication, and daytime dysfunction. Three studies used the Insomnia Severity Index (ISI)^17,22,30^, leading to a need for more specific information about this disturbance.

In the analysis of caregiver burden and stress, the studies used the Zarit Burden Interview Short Version (ZBI)^11,15,25,29,30,34^, the Stress Process Model of Caregiving (SPM)^8^, the Caregiver Burden Inventory (CBI)^33^, the Perceived Stress Scale (PSS)^16,28^, and the Relative Stress Scale (RSS)^5^.

### Caregiver sleep quality scores

The PSQI is a highly used self-reported questionnaire that evaluates sleep quality over the past month. The validity of PSQI is considered good, with a specificity of 86.5% and a sensitivity of 89.6%^35^. It analyzes seven sleep components that predict sleep quality: subjective sleep quality, sleep latency, sleep duration, sleep efficiency, sleep disturbances, use of sleep medication, and daytime dysfunction. A global score ≥5 predicts sleep disturbance.

Generally, the studies that used the PSQI to evaluate sleep quality expressed a global score above 5^1,3–5,11,14,21,25,31–33^. Only Liu et al.^34^ found a global score below 5. Unfortunately, two studies did not show the total score of PSQI^18,27^.

### Caregiver sleep disturbances

Caregivers of PLwD tend to spend less than 7 hours in bed, having high amounts of wake, impacting not only sleep quantity but also sleep quality^6^. Studies have found negative impacts in sleep latency^1,7,18,27,32^, sleep fragmentation^13,18,19,26^, shorter sleep duration^3,18^, and low subjective sleep quality^1,2,33^. In smaller proportions, they found insomnia^8^, use of medication^1,29^, daytime dysfunction^3^, and low sleep efficiency^1,6^ (Table 3).

**Table 3:** Objective measures.

Characteristic		% of articles citing the result
Pittsburgh sleep quality index	<5	3.7
	>5	40.7
Sleep patterns	Negative sleep latency	18.5
	Sleep fragmentation	14.8
	Shorter sleep duration	7.4
	Low subjective sleep quality	7.4
	Insomnia	3.7
	Daytime disfunction	3.7
	Use of medication	7.4
	Low sleep efficiency	7.4

### Caregiver burden, distress, and sleep

RSS positively correlates with PSQI^5^. Higher ZBI scores correlate with sleep disorders^15^ and shorter total sleep time^2,11,23,33^.

The association between role burden and maintenance insomnia was found in one study^8^. Jiménez-Gonzalo et al.^17^ found no correlation between caregiver sleep disturbances and significative scores on the ISI.

### Differences in caregiver sleep according to dementia type

Sleep disturbances are more prevalent in caregivers of individuals with neuropsychiatric symptoms, with caregivers presenting worse sleep quality when care recipients present disrupted behaviors^22^. More caregiving hours, a higher number of medical/nursing tasks, and more impairments in daily living implicate higher scores in the Level of Care Index (LCI), more sleep fragmentation, and greater sleep latency^14,18^. Also, recipients who presented sleep problems, such as difficulties in falling back asleep after waking up in the middle of the night, were associated with the caregiver having more sleep interruption^19^.

Recipients with Frontotemporal Dementia (FTD) and Dementia with Lewy Bodies (DLB) tend to present more neuropsychiatric symptoms demanding more care, leading to a higher burden and, consequently, more sleep problems^33^. Comparatively, DLB caregivers present more sleep problems than Alzheimer Disease (AD) caregivers. Other types of dementia were not analyzed^31^.

## DISCUSSION

This systematic review aimed to analyze the relationship between burden and sleep disturbances in caregivers of PLwD. Recent studies have found that the most impaired sleep components were sleep latency (the time the person takes to fall asleep after going to bed)^1,5,18,26,33^ and sleep fragmentation (sleep interruptions through the night)^13,18,19,26^. In addition, our findings corroborate the hypothesis that caregivers tend to spend less than seven hours in bed, leading to negative impacts on sleep quality and quantity, suggesting that this population has shorter sleep duration^3,18^, low subjective sleep quality^1,20^, maintenance insomnia^8^ and low sleep efficiency^1,6^.

Caregivers’ burden and distress positively correlate with sleep disturbances^5,11,15^. The perception of impairments in sleep quality is correlated with higher levels of burden^33^. Also, the daily stressor of caring for PLwD is related to reactivity in the hypothalamic-pituitary-adrenal (HPA) axis, leading to increased cortisol levels, which is related to less time in bed^12^. High cortisol levels negatively impact the Brain-Derived Neurotrophic Factor (BDNF), a neurotrophin related to sleep disorders^36^. The relationship between sleep and stress is mutual, once that the elevated rates of chronic stress^18^ and its physiological changes can be responsible for alterations in sleep patterns.

Results suggest differences between types of dementia and caregivers’ burden and sleep quality. For example, in FTD and DLB, the care recipient often presents agitation and sleep problems, which directly affects the quality of sleep and burden perception of caregivers^34^. Our findings also showed that caregivers who spend more hours caring for or performing more medical/nursing tasks have greater sleep fragmentation and latency^18^.

Sleep disorders are responsible for health problems such as high blood pressure^19^, higher Body Mass Index (BMI)^21^, high cortisol levels^12^, and psychiatric disorders^1^. Burden and sleep impairments often lead caregivers to present moderate depressive symptoms that lead to higher sleep latency, a greater number of wakes after sleeping, and low sleep efficiency^22,26,31^.

Cognitive impairments can be observed in this population^6^. For example, deficits in processing speed lead to negative impacts on care and generate problems such as errors in medication administration. Prolonged sleep deficits are associated to reduced clearance of Beta-amyloid and Thau^6^, leading to higher chances of developing cognitive impairment or dementia.

Sleep duration and fragmentation impact the coping strategies adopted by caregivers^18^ and their quality of life^29^. The perception of incapacity to rest can lead to inappropriate behaviors with the PLwD^15^ once the caregiver tends to lay their frustrations and irritability in the care recipient.

Female caregivers often report more mental health complaints and sleep problems. However, gender comparison is compromised by the fact that most caregivers are women, so this is a bias when comparing the groups. Also, most studies have fewer males in the sample, which can impact the results. Furthermore, caregivers of PLwD living in the community have more sleep deficits, leading to severe distress^2,5^, once that they usually cohabit with the care recipient^3^.

In conclusion, the results found positive correlations between caregivers’ sleep disturbances and burden, cognitive deficits, vulnerability to dementias, psychiatric disorders, and physical problems such as higher blood pressure, obesity, and high cortisol levels, predicting more significant mortality.

Understanding the factors related to caregiver sleep is essential to provide more health care to these populations and increase the quality of care given to PLwD. A telephone-based behavioral activation protocol showed that improved sleep quality leads caregivers to have a more positive perspective, reduced stress levels, and to be more open to accepting the opinions of health professionals^14^.

Better understanding and development of more effective interventions can lead to fewer health problems in caregivers and improve the quality of care provided to PLwD. Also, understanding and helping caregivers to improve their sleep quality and consequently their quality of life can reduce government financial expenditure on health systems, as there will be a healthier population.
